# Model predictive control of a brushless cascadedoubly-fed induction generator with torque regulation for stand-alone applications

**DOI:** 10.1016/j.heliyon.2024.e40449

**Published:** 2024-11-15

**Authors:** Hatam Abdolrahimi, Davood Arab Khaburi

**Affiliations:** Iran University of Science and Technology, Iran

## Abstract

In this paper, a novel control method for Brushless Cascade Doubly-Fed Induction Generator (BCDFIG) control is proposed by integrating the Model Predictive Control (MPC) and the Direct Torque Control (DTC) methods. In previous studies, the DFIG's torque control has been performed, using different methods to improve power production. Here, direct torque control, assisted by model predictive control, is used to maintain output voltage during a change in wind speed and load. To reduce switching loss in the inverter, the model predictive unit selects the optimized switching vector and sends it to the DTC unit. According to a predefined switching table in each phase interval, the proper switching sequence for controlling the flux and torque for producing a constant output voltage will be selected. This method can be useful for wind farms and everywhere we need constant voltage, especially in stand-alone applications. Also, this proposed method can be used in grid-connected networks for injecting power into the grid or controlling reactive power. For evaluating this control method, in the first step, the BCDFIG setup is simulated in MATLAB software. Then an experimental setup has been realized in the lab to perform some tests. At the end, the obtained experimental results were investigated.

**Table:** Nomenclature

Power machine and control machine	PW_M, CNT_M
PW_M and its rotor angular frequency	$\omega_{pw},\omega_{r\_pw}$
CNT_M and its rotor angular frequency	$\omega_{cnt},\omega_{r\_cnt}$
Sampling time	$T_{s}$
Voltage, current and flux	V,i,λ
Resistance and Inductance	R, L
Sample number	k
PW_M stator, CNT_M stator and rotor	s_pw,s_cnt,r
PW_M rotor, CNT_M rotor	r_pw,r_cnt
Leakage, PW_M and CNT_M mutual Inductance	l,m_pw,m_cnt
d-q rotating frame	d, q
Local load	$R_{L}$
BCDFIG's torque	$\tau_{BCDFIG}$
Nominal torque of the BCDFIG	$\tau_{N\_BCDFIG}$
reference torque of the BCDFIG	$\tau_{ref}$

## 1 Introduction

With the growing demand for electrical energy, particularly in developed countries, it becomes necessary to develop energy production units. These units typically use fossil fuels to produce electrical power, but due to the global drive to reduce CO2 production, the source of electrical power generation is expected to shift to clean sources. Wind turbines are one of the common sources of electrical energy production.

One of the usual electrical machines for wind turbines in recent decades has been double-fed induction machines, which could be controlled with an automatic cascade structure [[1], [2], [3], [4], [5]]. DFIG needs regular maintenance and repairs because of its brushes, slip rings, and its suboptimal performance during grid voltage drops [[6], [7], [8], [9]]. A considerable amount of research has been carried out on doubly-fed brushless machine generators to take advantage of DFIG and solve its existing problems. As a result, brushless doubly-fed induction generators (BDFIGs) have been explored as potential future generators for wind turbines [[10], [11], [12], [13], [14]].In this structure, two coils are placed on the generator stator, and each of them has a particular task. One of these coils, called the control coil, is responsible for controlling power and torque by changing the amount of flux in the air gap. The second coil is referred to as the power coil, which plays a role in generating mechanical energy when operating in motor mode and electrical energy when functioning in generator mode [[15], [16], [17], [18], [19], [20]]. Despite all its advantages, this method cannot be used everywhere due to its structural and control complexity. For this reason, in this manuscript, another structure of DFIG generators in which the power and control machine are externally coupled, known as BCDFIG, is used, which is easily available and applicable in this industry.

Wind speed usually changes, so the output voltage may change over time. By changing the output voltage, the energy injection into the network can be interrupted [21]. Many papers have examined various control strategies to maintain output voltage as wind speed changes in an attempt to resolve this issue [22,23]. Some of studies include current control, power control, and torque control. In addition to the mentioned methods, the use of PI controllers, which have a long history in the field of control systems, is also widely used [24,25]. One option to control the circuit using the mentioned controllers is to use vector controllers based on PI loops. This method may not be economically attractive due to the need for heavy calculations and the need for multiple sensors [26].

Another method mentioned in the articles is direct torque control using voltage controlled inverters. In this method, the switching frequency changes continuously to control different speeds of the generator. Nowadays, direct torque control is one of the best-known control methods, especially in stand-alone generators where the terminal voltage needs to be controlled.

In [27], direct torque control is used to control the terminal voltage in case of unbalanced grid voltage. This method is useful for synchronous speed changes of 25 %. A modified DTC torque control method for DFIGs with model predictive control is presented in Ref. [28].In this method, the reference flux and voltage are obtained by the DFIG model, then the torque control will be carried out with reference values [29,30]. focused on the network connection control method.

The use of predictive controllers using power control actuators is also one of the newest methods to control DFIG generators [31]. The basic idea of predictive control is to predict the behavior of the system at time t+1, in time t, using the required mathematical model of the system. The ultimate goal of this control system is to minimize the error or deviation between the target value and the existing value by predicting the future response of the system and applying the necessary control commands and optimization algorithms. One of the main advantages of this system is that, through the prediction of the future response of the system based on the available information, noise and interference that momentarily or intermittently enter the system will be eliminated. Recently, electricity and renewable energy systems have used predictive control methods. The relationship between the voltage derivative of the power machine stator in the dq frame forms the foundation for MPC modeling [[32], [33], [34], [35], [36], [37], [38]].

Various studies suggest different forms of DTC and MPC methods for BCDFIG systems. This paper proposes a control strategy that integrates an MPC unit with the DTC method to maintain stable amplitude and frequency of output voltages in a BCDFIG, even with variations in load and generator shaft speed in stand-alone operation. In this model predictive direct torque control (MPDTC) method, the BCDFIG is controlled by the DTC method, and the optimal reference torque and flux are derived from a mathematical model in an MPC unit. The MPC unit evaluates the rated torque and actual torque of the generator as well as the system noise. Then, according to the cost function, it predicts the optimal torque reference in the next step. According to the optimal reference torque, the generator output voltage and the load output current, the DTC unit determined the appropriate voltage vector to be applied to the stator of the control machine. The effectiveness of the proposed method was evaluated based on practical results. Finally, some simulations and experiments were carried out. The obtained results show that the MPDTC has better efficiency and faster dynamic responses than other common control methods.

To explain the control method, first of all, the considered model for the generator is fully explained, and all primary relationships are expressed and simulated. Then, the proposed control method is modeled using MATLAB software and applied to the generating model. the advantages of this method compared to other control methods will be discussed.

## 2 Model of proposed system

Firstly, to study the proposed control method, the mathematical equations governing the dynamic behavior of electrical machines will be explained.

**Fig. 1:**
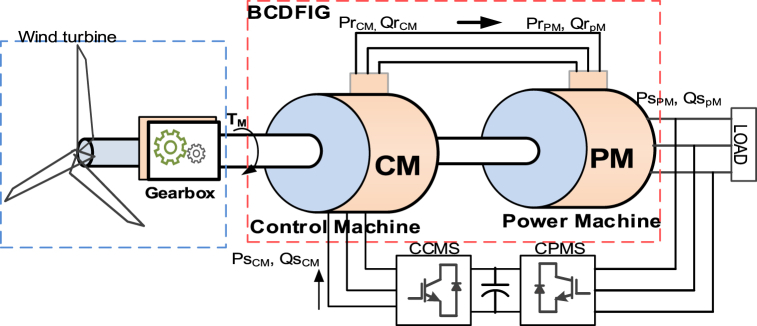
A schematic representation of the BCDFIG structure [33].

**Fig. 2:**
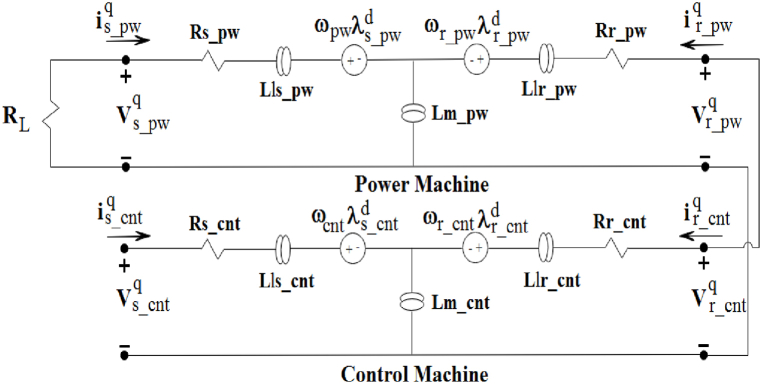
The q-axis equivalent circuit of the BCDFIG [33].

**Fig. 3:**
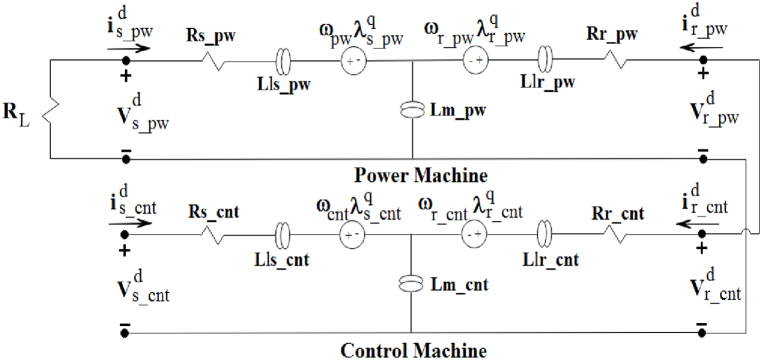
The d-axis equivalent circuit of the BCDFIG [33].

As shown in Fig. 1, the proposed control structure includes two electrical machines, one of which is the controller and the other is the power generator. The two electrical machines are interconnected both mechanically and electrically. The state models of electrical machines in the dq coordinate system are as described in Fig. 2, Fig. 3.

(1) $$\begin{array}{l} \left\{ \begin{array}{l} L_{s\_pw}=L_{ls\_pw}+L_{m\_pw}\\ L_{s\_cnt}=L_{ls\_cnt}+L_{m\_cnt} \end{array}\right.\\ \left\{ \begin{array}{l} L_{r\_pw}=L_{lr\_pw}+L_{m\_pw}\\ L_{r\_cnt}=L_{lr\_cnt}+L_{m\_cnt} \end{array}\right. \end{array}$$

Equation (1) provides the variables that represent the total main and leakage inductances of the power and control machines. Equation (2) presents the flux equations for the rotor and stator in power and control machines within the d-q system.

(2) $$\begin{array}{l} \left\{ \begin{array}{l} \lambda_{s\_pw}^{dq}=L_{s\_pw}i_{s\_pw}^{dq}+L_{m\_pw}i_{r\_pw}^{dq}\\ \lambda_{r\_cnt}^{dq}=L_{r\_cnt}i_{r\_cnt}^{dq}+L_{m\_cnt}i_{s\_cnt}^{dq} \end{array}\right.\\ \left\{ \begin{array}{l} \lambda_{r\_pw}^{dq}=L_{r\_pw}i_{r\_pw}^{dq}+L_{m\_pw}i_{s\_pw}^{dq}\\ \lambda_{s\_cnt}^{dq}=L_{s\_cnt}i_{s\_cnt}^{dq}+L_{m\_cnt}i_{r\_cnt}^{dq} \end{array}\right. \end{array}$$

Additionally, equations (3), (4), (5), (6) can be used to determine the rotor and stator voltages in power and control systems.

(3) $$v_{s\_pw}^{dq}=R_{s\_pw}i_{s\_pw}^{dq}+\frac{d\lambda_{s\_pw}^{dq}}{dt}\pm \omega_{pw}\lambda_{s\_pw}^{dq}$$

(4) $$v_{r\_pw}^{dq}=R_{r\_pw}i_{r\_pw}^{dq}+\frac{d\lambda_{r\_pw}^{dq}}{dt}\pm \omega_{r\_pw}\lambda_{r\_pw}^{dq}$$

(5) $$v_{r\_cnt}^{dq}=R_{r\_cnt}i_{r\_cnt}^{dq}+\frac{d\lambda_{r\_cnt}^{dq}}{dt}\pm \omega_{r\_cnt}\lambda_{r\_cnt}^{dq}$$

(6) $$v_{s\_cnt}^{dq}=R_{s\_cnt}i_{s\_cnt}^{dq}+\frac{d\lambda_{s\_cnt}^{dq}}{dt}\pm \omega_{cnt}\lambda_{s\_cnt}^{dq}$$

Previous studies that considered the rotor to be integrated assumed the rotor voltage of the BCDFIG to be zero, which $v_{r\_pw}^{dq}=v_{r\_cnt}^{dq}$ is considered in this paper.

The voltages and currents of the rotors in a BCDFIG are equal because of the electrical coupling between them.

(7) $$Vr_{pw}=Vr_{cnt},ir_{pw}=ir_{cnt}$$

Hence, the equations of (4) and (5) can be considered equal. The equations for the stator voltage of power machines can be represented in the dq reference frame using equation (8).

(8) $$\begin{array}{l} \frac{dv_{s\_pw}^{q}}{dt}=\frac{R_{L}}{L_{m\_pw}}[R_{r}i_{r\_pw}^{q}+L_{r}\frac{di_{r\_pw}^{q}}{dt}\\ \begin{array}{ccc} & & \begin{array}{cccc} & & & -L_{m\_cnt}\frac{di_{s\_cnt}^{q}}{dt}-\omega_{r\_pw}\left(\lambda_{r\_cnt}^{d}-\lambda_{r\_pw}^{d}\right)] \end{array} \end{array} \end{array}$$

In the rotor winding of PM and CM machines, it is important to note that there is no voltage source. This results in the rotor voltage being zero. By substituting equations (7), (3), the rotor current of the power machine can be obtained as shown in equation (9).

$$\begin{array}{cc} j_{1}=1+\frac{R_{s\_pw}}{R_{L}} & ,j_{2}=-L_{m\_pw}+\frac{L_{r}L_{s\_pw}}{L_{m\_pw}} \end{array},\text{so,}$$

(9) $$\begin{array}{l} \frac{di_{r\_pw}^{q}}{dt}=\frac{1}{j_{2}}[-j_{1}v_{s\_pw}^{q}-\frac{L_{s\_pw}}{L_{m\_pw}}R_{r}i_{r\_pw}^{q}+\frac{L_{s\_pw}L_{m\_cnt}}{L_{m\_pw}}\frac{di_{s\_cnt}^{q}}{dt}\\ \begin{array}{cccc} & & & +\frac{L_{s\_pw}}{L_{m\_pw}}\omega_{r\_pw}\left(\lambda_{r\_cnt}^{d}-\lambda_{r\_pw}^{d}\right)+\omega_{pw}\lambda_{s\_pw}^{d}] \end{array} \end{array}$$

By substituting Equation (9) into Equation (6) and assuming $j_{3}=L_{s\_cnt}-\frac{L_{m\_cnt}^{2}L_{s\_pw}}{j2L_{m\_pw}}$, the result is presented in Equation (10).

(10) $$\begin{array}{l} \frac{di_{s\_cnt}^{q}}{dt}=\frac{1}{j_{3}}[v_{s\_cnt}^{q}-R_{s\_cnt}i_{s\_cnt}^{q}-\frac{j_{1}}{j_{2}}L_{m\_cnt}v_{s\_pw}^{q}\\ \begin{array}{cccc} & & & -\frac{L_{m\_cnt}}{j_{2}}\frac{L_{s\_pw}}{L_{m\_pw}}R_{r}i_{r\_pw}^{q} \end{array}\\ \begin{array}{cccc} & & & +\frac{L_{m\_cnt}}{j_{2}}\frac{L_{s\_pw}}{L_{m\_pw}}\omega_{r\_pw}\left(\lambda_{r\_cnt}^{d}-\lambda_{r\_pw}^{d}\right) \end{array}\\ +\frac{L_{m\_cnt}}{j_{2}}\omega_{pw}\lambda_{s\_pw}^{d}-\omega_{cnt}\lambda_{s\_cnt}^{d}] \end{array}$$

By continuing the techniques applied to the d-axis, the rotor and stator equations in power and control machines can be determined based on Equation (6).

(11) $$\begin{array}{l} \frac{dv_{s\_pw}^{d}}{dt}=\frac{R_{L}}{L_{m\_pw}}[R_{r}i_{r\_pw}^{d}+L_{r}\frac{di_{r\_pw}^{d}}{dt}-L_{m\_cnt}\frac{di_{s\_cnt}^{d}}{dt}\\ \begin{array}{cccc} & & & \begin{array}{cc} & +\omega_{r\_pw}\left(\lambda_{r\_cnt}^{q}-\lambda_{r\_pw}^{q}\right)] \end{array} \end{array} \end{array}$$

(12) $$\begin{array}{l} \frac{di_{r\_pw}^{d}}{dt}=\frac{1}{j_{2}}[-j_{1}v_{s\_pw}^{d}-\frac{L_{s\_pw}}{L_{m\_pw}}R_{r}i_{r\_pw}^{d}+\frac{L_{s\_pw}L_{m\_cnt}}{L_{m\_pw}}\frac{di_{s\_cnt}^{d}}{dt}\\ -\frac{L_{s\_pw}}{L_{m\_pw}}\omega_{r\_pw}\left(\lambda_{r\_cnt}^{q}-\lambda_{r\_pw}^{q}\right)-\omega_{pw}\lambda_{s\_pw}^{q}] \end{array}$$

(13) $$\begin{array}{l} \frac{di_{s\_cnt}^{d}}{dt}=\frac{1}{j_{3}}[v_{s\_cnt}^{d}-R_{s\_cnt}i_{s\_cnt}^{d}-\frac{j_{1}}{j_{2}}L_{m\_cnt}v_{s\_pw}^{d}\\ \begin{array}{cccc} & & & \begin{array}{cc} & -\frac{L_{m\_cnt}}{j_{2}}\frac{L_{s\_pw}}{L_{m\_pw}}R_{r}i_{r\_pw}^{d} \end{array} \end{array}\\ \begin{array}{cccc} & & & \begin{array}{cc} & -\frac{L_{m\_cnt}}{j_{2}}\frac{L_{s\_pw}}{L_{m\_pw}}\omega_{r\_pw}\left(\lambda_{r\_cnt}^{q}-\lambda_{r\_pw}^{q}\right) \end{array} \end{array}\\ \begin{array}{cccc} & & & \begin{array}{cc} & -\frac{L_{m\_cnt}}{j_{2}}\omega_{pw}\lambda_{s\_pw}^{q}+\omega_{cnt}\lambda_{s\_cnt}^{q}] \end{array} \end{array} \end{array}$$

## 3 MPC modeling

The forward approximation of the Euler derivative defines the discrete-time model according to the continuous-time equations in order to simplify the model system [39]. The Euler derivative can be approximated by using equation (14).

(14) $$\frac{di}{dt}=\frac{i\left(k\right)-i\left(k-1\right)}{T_{s}}$$

Upon substituting equations (9), (12) into equations (7), (11), the following expressions are derived.

(15) $$\begin{array}{l} v_{s\_pw}^{q}\left(k+1\right)=v_{s\_pw}^{q}\left(k\right)+\frac{T_{s}R_{L}}{L_{m\_pw}}[R_{r}i_{r\_pw}^{q}\left(k\right)\\ \begin{array}{cccc} & & & \begin{array}{ccc} & & +L_{r}i_{r\_pw}^{q}\left(k+1\right) \end{array} \end{array}-L_{m\_cnt}i_{s\_cnt}^{q}\left(k+1\right)\\ \begin{array}{cccc} & & & \begin{array}{ccc} & & -\omega_{r\_pw}\left(k\right)\left[\lambda_{r\_cnt}^{d}\left(k\right)-\lambda_{r\_pw}^{d}\left(k\right)\right]] \end{array} \end{array} \end{array}$$

(16) $$\begin{array}{l} v_{s\_pw}^{d}\left(k+1\right)=v_{s\_pw}^{d}\left(k\right)+\frac{T_{s}R_{L}}{L_{m\_pw}}[R_{r}i_{r\_pw}^{d}\left(k\right)\\ \begin{array}{cccc} & & & \begin{array}{ccc} & & +L_{r}i_{r\_pw}^{d}\left(k+1\right)-L_{m\_cnt}i_{s\_cnt}^{d}\left(k+1\right) \end{array} \end{array}\\ \begin{array}{ccc} & & \begin{array}{cc} & \begin{array}{cc} & +\omega_{r\_pw}\left(k\right)\left[\lambda_{r\_cnt}^{q}\left(k\right)-\lambda_{r\_pw}^{q}\left(k\right)\right]] \end{array} \end{array} \end{array} \end{array}$$

The Euler approximation, along with equations (9), (10), (12), (13), provides a straightforward approach to determining the instantaneous values for step (k+1) as represented in equations (15), (16). The function of this method relies on applying the above voltage vectors to equations (7), (8), (9), (10), (11), (12), (13). The cost function presented in equation (17) is evaluated for every one of the seven potential switching states [40].

(17) $$g=\left|v_{s\_pw\_ref}^{q}-v_{s\_pw}^{q}\left(sw\right)\right|+\left|v_{s\_pw\_ref}^{d}-v_{s\_pw}^{d}\left(sw\right)\right|$$

SW represents the switching mode, which can take values from 0 to 7.

**Fig. 4:**
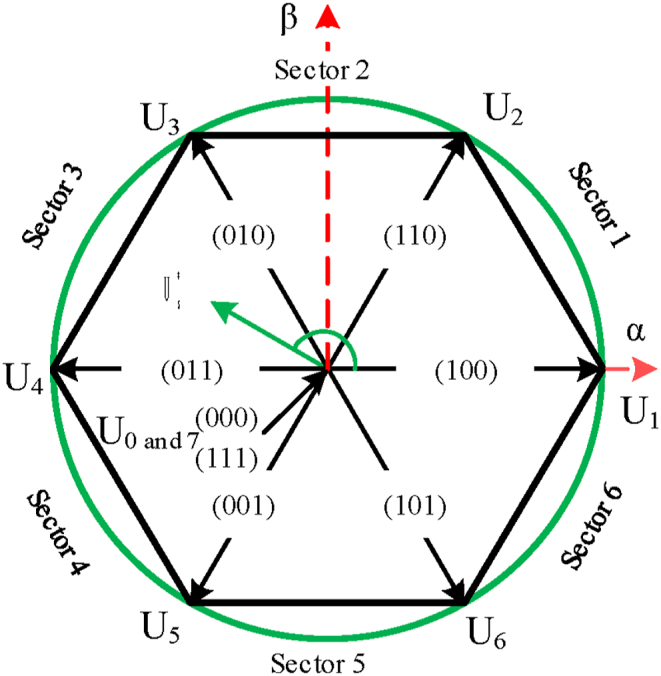
Voltage vectors used in the MPC [33].

Fig. 4 shows the eight different switching modes (U1, U2, …, U7) that result from the MPC method. In every step, a vector is chosen that minimizes the cost function and is then applied to the stator of the control machine in the next step [33].

## 4 MPDTC control method

In the DTC control method, although there is a rapid response to changes in control parameters due to fast switching, there may be switching vectors that do not play a role in controlling the parameters of flow and torque and only increase losses in the inverter [[41], [42], [43]]. By adding MPC control to the DTC, an attempt was made to increase the accuracy of the control circuit and reduce the number of losses in the control inverter.

The working method is as follows: firstly, using the model and cost function of the MPC unit, the optimal value of the switching vector is selected and transferred to the control unit. This value is based on the control parameter prediction based on the MPC model. This value makes the DTC unit smarter to avoid zero switching. By using this method, in addition to reducing the maintenance costs of wind turbine inverters, losses can also be reduced.

To control the torque and flux in each section, the position of the control vector is first determined, and based on this, the appropriate switching state to change the torque and flux will be selected from the lookup table.

In Table 1, 110 means that the first and second legs should be connected to positive voltage and the last leg should use auxiliary switches to reach zero voltage. " $H_{Te}$" stands for the direction of torque change. For example, a value of +1 indicates an increase in torque, while a value of −1 signifies a decrease in torque. Similarly, " $H_{\varphi }$" represents the direction of flux change, with values of +1, −1, and 0 indicating an increase, decrease, and no change in state, respectively.

**Table 1:** Switching look-up table.

$H_{\varphi }$	$H_{Te}$	S(1)	S(2)	S(3)	S(4)	S(5)	S(6)
1	1	110	010	011	001	101	100
	0	000	000	000	000	000	000
	−1	101	100	110	010	011	001
−1	1	010	011	001	101	100	110
	0	000	000	000	000	000	000
	−1	001	101	100	110	010	011

**Fig. 5:**
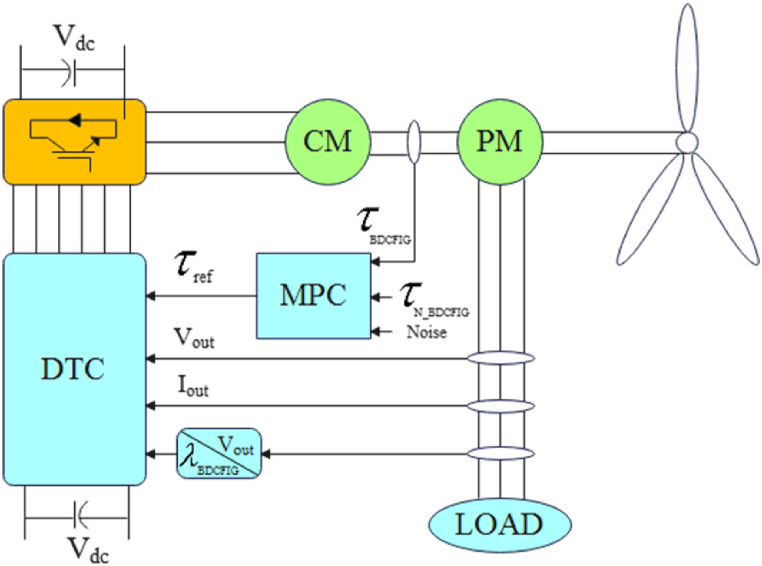
Block diagram of the control strategy proposed for the BCDFIG system.

We can control any voltage change across the power machine using the predictive section and DTC section. A constant voltage terminal can be obtained by controlling electrical flow and torque, even when the load or shaft speed changes. The block diagram of the proposed control method is shown in Fig. 5.

## 5 Simulation and practical implementation

The equations in the previous section are implemented in MATLAB, Simulink and M-File to show the efficiency and performance of the proposed method. Table 2 lists the specifications of the BCDFIG prototype employed in the simulation study. To generate speed, a DC machine—known as the Prime Mover—is used instead of a wind turbine. An inverter provides controllable power for the stator of the control machine, produced by the STM32H743II.

**Table 2:** Parameters used in simulation.

Specifications	Power Machine	Control Machine
Stator resistance	1.83	2.55
Rotor resistance	2.42	2.57
Stator leakage inductance (H)	1.92	2.66
Rotor leakage inductance (H)	1.92	2.66
Magnetic inductance (H)	58.43	156.63
Rated power (kw)	3	1
Number of poles	4	4

**Fig. 6:**
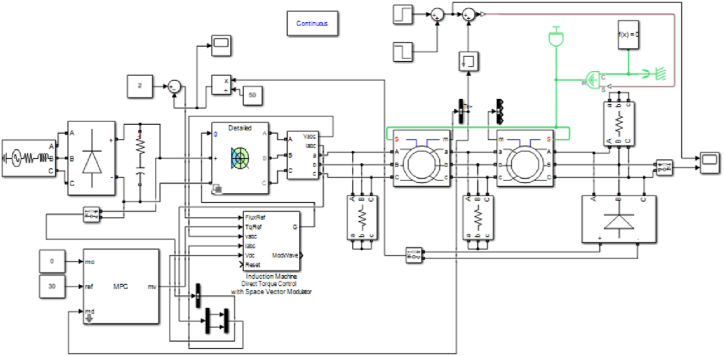
Simulink model of BCDFIG.

In Fig. 6, the simulated model has been shown in MATLAB 2018a. The parameters of the machines have been set according to Table 2.

**Fig. 7:**
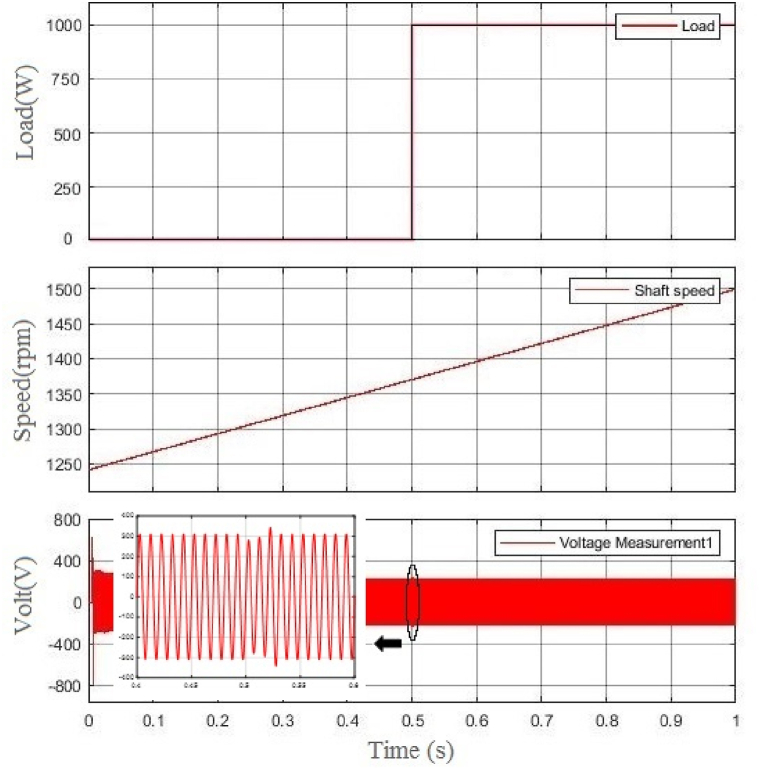
From top, the first figure is the change of load, the second one is the change of BCDFIG shaft speed, and the last one is the output voltage at the power machine.

The simulated scenario is that the load changes from 0 p.u. to 1 p.u. at 0.5s while the shaft speed increases; as Fig. 7 shows, the voltage's amplitude doesn't change significantly.

**Fig. 8:**
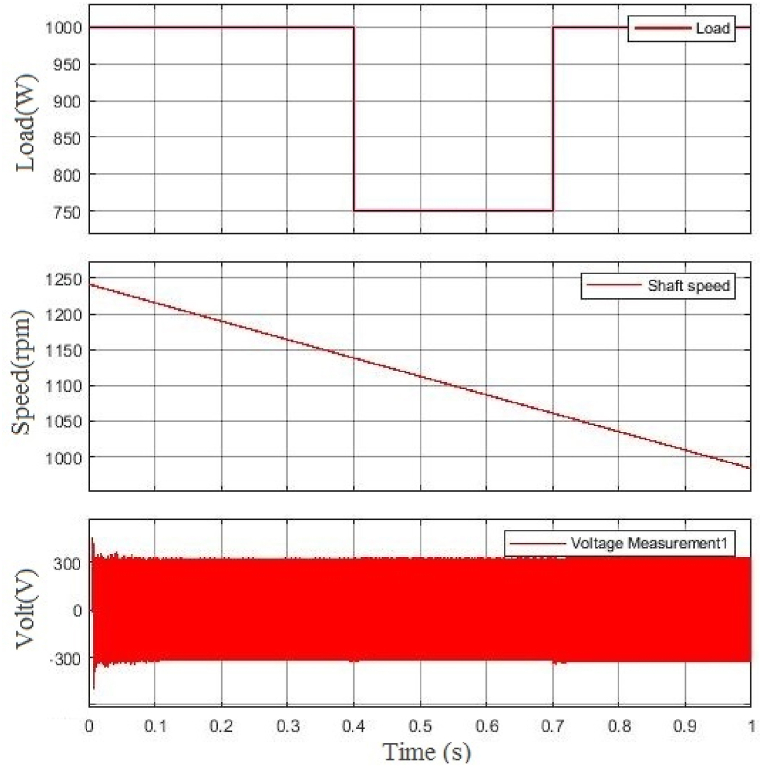
From top, the first figure is the change of load, the second one is the change of BCDFIG shaft speed, and the last one is the output voltage at the power machine.

In the second simulation scenario, when the shaft speed decreases, the load changes from 1 p.u. to 0.75 p.u. in 0.4s and then changes back to 1 p.u. in 0.7s. Fig. 8 shows how the generator's voltage doesn't change. Thus, the voltage amplitude has remained unchanged since then, while the load or shaft speed has changed.

**Fig. 9:**
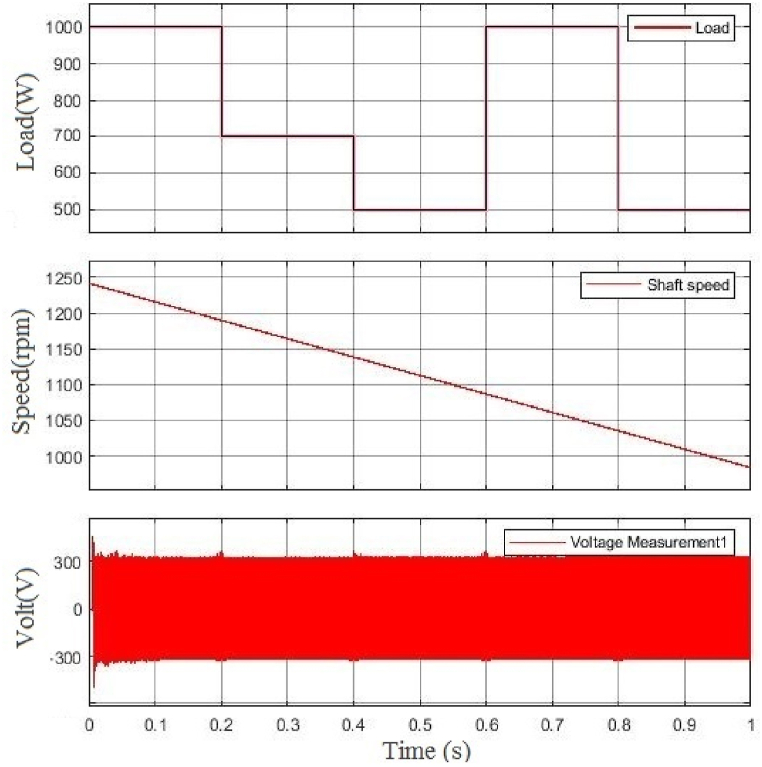
From top, the first figure is the change of load, the second one is the change of BCDFIG shaft speed, and the last one is the output voltage at the power machine.

In the last simulation scenario, the load changes continuously while the speed of the generator shaft is decreasing. Generator voltage changes are shown in Fig. 9. This scenario illustrates that the proposed method effectively maintains the amplitude and frequency of the BCDFIG output voltages in standalone generation systems, even in the presence of sudden variations in load current and mechanical fluctuations in generator shaft speed.

## 6 Experimental results

**Fig. 10:**
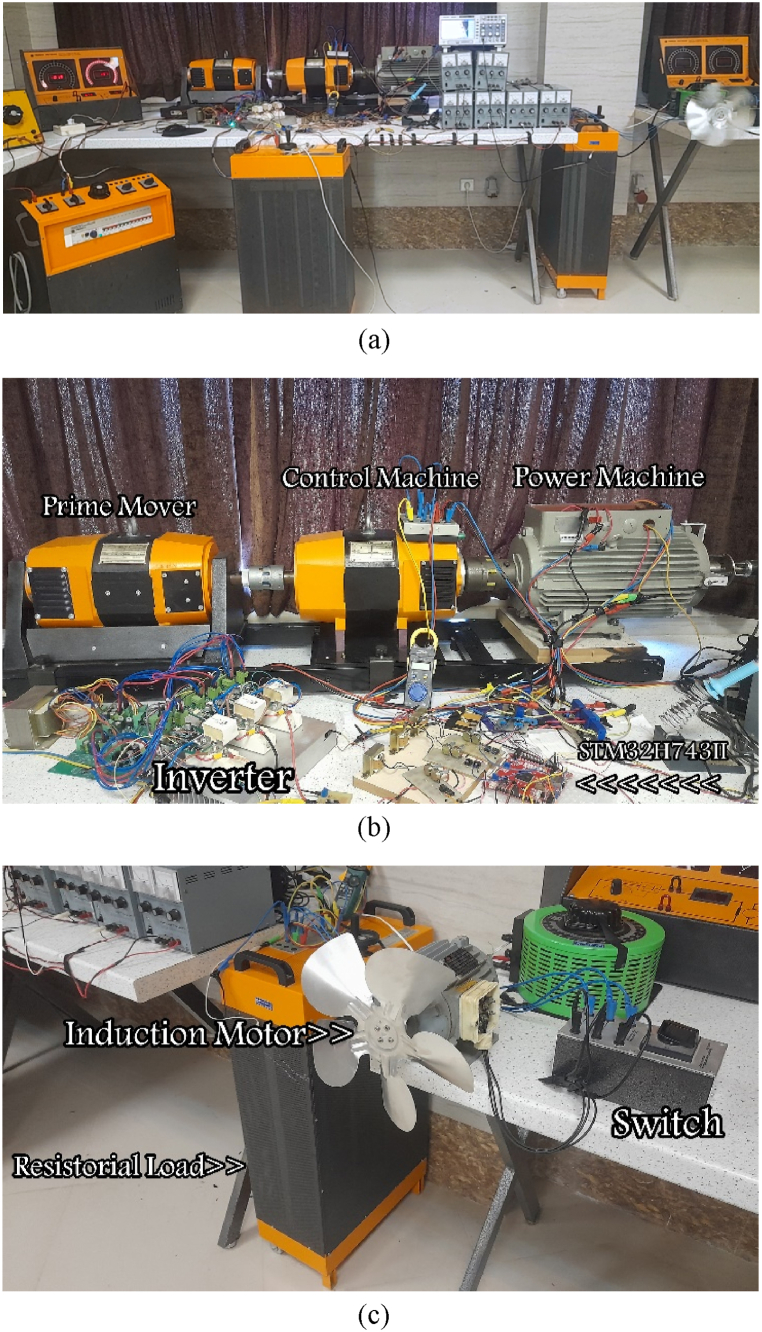
The set-up of the BCDFIG. (a) Overview of set-up; (b) BCDFIG components; (c) Load of BCDFIG.

A testbed for the BCDFIG prototype, illustrated in Fig. 10, is suggested to validate the simulation outcomes of the theoretically proposed method by implementing the control strategy. Fig. 10(a) shows the setup overview, Fig. 10(b) shows the BCDFIG components, and Fig. 10(c) shows the BCDFIG load. The power configuration involves coupling two DFIG machines in cascade configurations. Additionally, to give the starting speed, a DC machine with a variable speed shaft is coupled to the BCDFIG. The stator of the control machine is supplied by the two-level back-to-back converter, whereas the stator of the power machine directly powers the local load, and the speed of the BCDFIG shaft is rotated by the control machine. and the shaft of BCDFIG is rotated by a DC machine.

To evaluate the steady-state performance of the proposed control strategy for the BCDFIG, a series of tests were conducted.

**Fig. 11:**
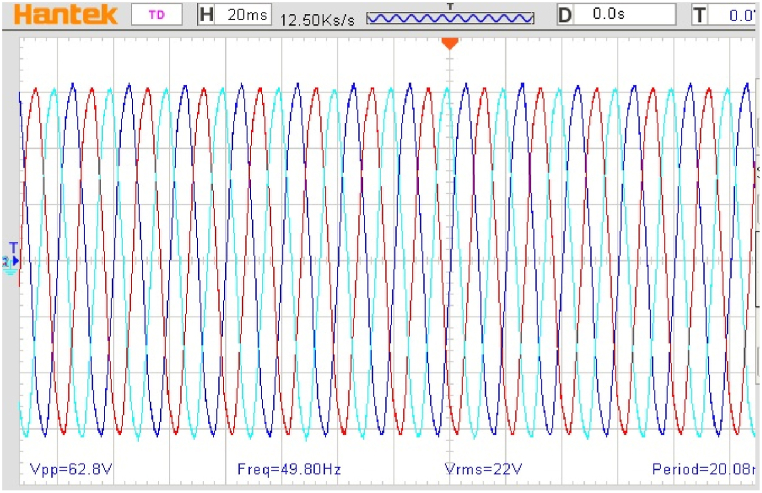
The voltage at the stator of the PM.

Fig. 11 of the first test shows the stator voltage of the power machine, operating at full load, with the voltage zoomed in without any distortion, validating the quality of the energy generated by the power machine.

**Fig. 12:**
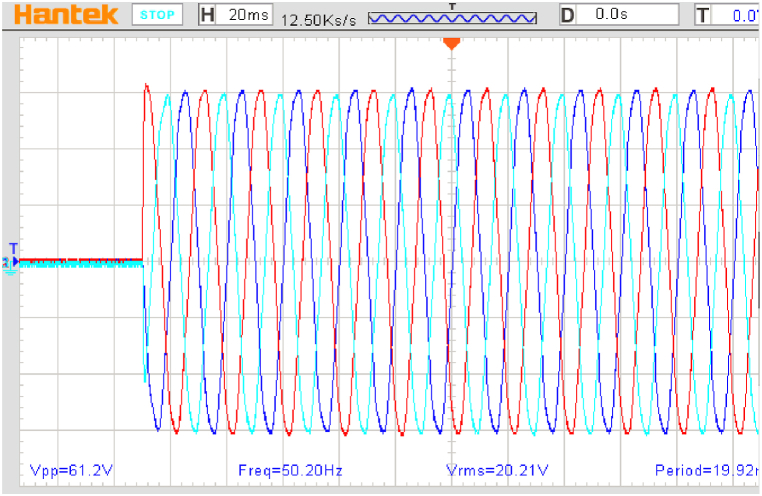
The BCDFIG stator voltage for an output load step change of 100 %.

A step change in output load from 0 % to 100 % was performed to investigate the proposed system's dynamic responsiveness, corresponding to the simulation section (Fig. 12).

**Fig. 13:**
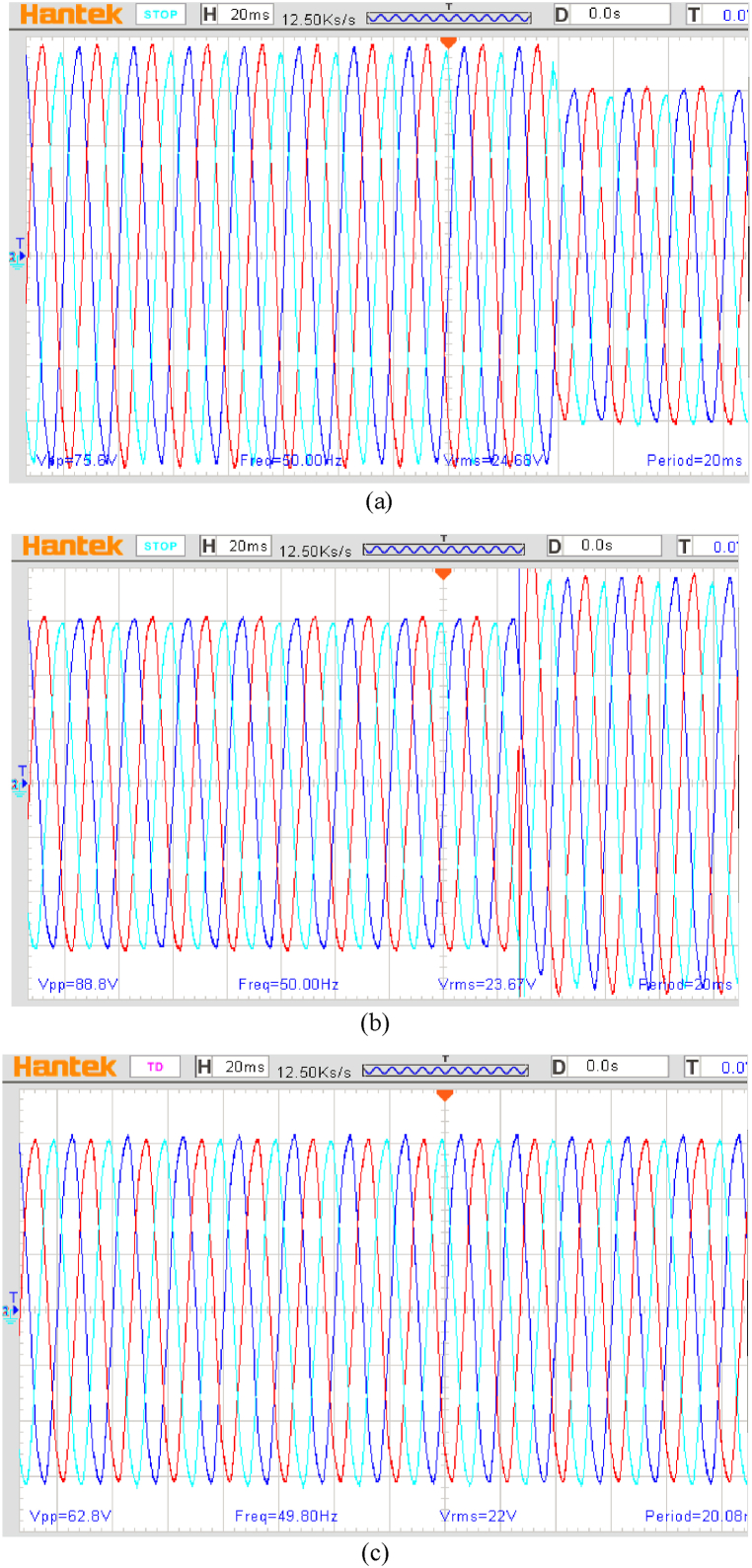
Output voltage of the BCDFIG in changes of load and speed of generator shaft. (a) 25 % increase in BCDFIG load; (b) 25 % reduction in BCDFIG load; (C) output voltage of the BCDFIG.

In the end, in order to validate the proposed control method, the generator load decreased by 1 p.u. at 0.75 p.u (Fig. 13(a)) and then increased again to 1 p.u (Fig. 13(b)). In all of this, the generator shaft speed decreases. Fig. 13(c) shows that the output voltage of BCDFIG remains constant during this variation period.

Therefore, the wide bandwidth MPDTC tracks the references exactly even for small errors with very high accuracy. However, alternative control strategies, such as PI, often lead to significant control errors due to limitations in bandwidth. The experimental results indicate that the MPDTC demonstrates rapid performance and remains robust under variations in shaft speed and load. Table 3 compares the BCDFIG with MPDTC efficiency to other control methods with an efficiency level above 93 %.

**Table 3:** Evaluation of the Method Controller's effectiveness.

Method Controller	Efficiency (%)
PID [44]	87
SMC With DPC [45]	90
MPC with PTC [44]	90
LQG [44]	92
MPDTC (Proposed)	93

## 7 Conclusion

In this paper, a new method was studied to avoid output voltage fluctuations and reduce zero vector switching in the inverter based on changes in load and shaft speed. In this method, a model predictive block calculates the future value of flux and torque due to variations in load and speed and then estimates the appropriate switching vector. In the next step, the derived references values of flux and torque are transmitted to the DTC unit to control the Control Machine. In the DTC unit, suitable switching states are selected to bring the existing value closer to the reference value and the estimated vector. The simulation and experimental results showed that in different situations, by changing the load and speed of the shaft, the output voltage remained constant, and this control method could control the situation in the case of blushless cascade doubly feed machines.

## CRediT authorship contribution statement

**Hatam Abdolrahimi:** Writing – original draft. **Davood Arab Khaburi:** Writing – review & editing.

## Data availability

The datasets used and/or analysed during the current study available from the corresponding author on reasonable request.

## Declaration of Competing Interest

The authors declare that they have no known competing financial interests or personal relationships that could have appeared to influence the work reported in this paper.
